# COVID- 19 Following Acute Psychosis: Case series report

**DOI:** 10.1192/j.eurpsy.2022.1363

**Published:** 2022-09-01

**Authors:** A. Hrnjica, Š. Šarkić-Bedak, I. Lokmić- Pekiċ

**Affiliations:** 1 Psychiatric hospital Sarajevo, Women Department, Sarajevo, Bosnia and Herzegovina; 2 Psychiatric hospital Sarajevo, Intensive Care Department, Sarajevo, Bosnia and Herzegovina

**Keywords:** brief psychotic disorder, Covid-19, acute psychosis

## Abstract

**Introduction:**

Coronavirus disease (COVID-19) could result in various medical consequences. Clinical manifestations are diverse and range from asymptomatic or mild, fever-like symptoms, to more severe and life-threatening complications. Although the clinical presentation was initially dominated by respiratory symptoms, psychiatric symptoms and sequelae have been reported in COVID- 19 patients and convalescents.

**Objectives:**

To describe four clinical case reports of patients admitted to the inpatient unit of Psychiatric Hospital Sarajevo (PHS) with acute onset of psychosis in the recovery stage of COVID-19.

**Methods:**

In this case series we report cases of 4 patients, all female, mean age 53.25 years, hospitalized in PHS during the third wave of the COVID-19 pandemic. All developed psychotic symptoms in the recovery phase of COVID-19. None had a previous history of psychiatric disturbances of any kind. All patients were diagnosed with brief psychotic disorder (BPD), according to DSM 5 criteria for BPD.

**Results:**

COVID-19 affects various organ systems, including the brain, with variable symptoms based on disease severity. Psychotic features have been observed as well. The pathophysiology and direct biological effects of the disease are not fully understood. COVID-19 patients and convalescents can develop psychotic symptoms as a consequence of multiple concurrent factors. Several proposed mechanisms include direct central nervous system infiltration, cytokine network dysregulation, peripheral immune cell transmigration, and post-infectious autoimmunity [1], treatments used to manage the infection, and psychosocial stress.

**Conclusions:**

Clinicians need to be aware of possible psychotic manifestations in COVID-19 patients and survivors. Long-term follow-up is warranted to provide efficient patient care.

**Disclosure:**

No significant relationships.

